# Long-Term Outcomes of Stereotactic Radiosurgery for Trigeminal, Facial, and Jugular Foramen Schwannoma in Comparison with Vestibular Schwannoma

**DOI:** 10.3390/cancers13051140

**Published:** 2021-03-07

**Authors:** Yuki Shinya, Hirotaka Hasegawa, Masahiro Shin, Takehiro Sugiyama, Mariko Kawashima, Atsuto Katano, Akinori Kashio, Kenji Kondo, Nobuhito Saito

**Affiliations:** 1 Department of Neurosurgery, The University of Tokyo Hospital, Tokyo 113-8655, Japan; hirohasegawa-tky@umin.ac.jp (H.H.); SHIN-NSU@h.u-tokyo.ac.jp (M.S.); mrkawashima-tky@umin.ac.jp (M.K.); nsaito-tky@umin.net (N.S.); 2 Department of Neurologic Surgery, Mayo Clinic, Rochester, MN 55905, USA; 3 Diabetes and Metabolism Information Center, Research Institute, National Center for Global Health and Medicine, Tokyo 162-8655, Japan; tsugiyama-tky@umin.ac.jp; 4 Department of Health Services Research, Faculty of Medicine, University of Tsukuba, Ibaraki 305-8575, Japan; 5 Department of Radiology, The University of Tokyo Hospital, Tokyo 113-8655, Japan; KATANO-RAD@h.u-tokyo.ac.jp; 6 Department of Otorhinolaryngology, The University of Tokyo Hospital, Tokyo 113-8655, Japan; kashioa-tky@umin.ac.jp (A.K.); kondok-tky@umin.ac.jp (K.K.)

**Keywords:** intracranial schwannomas, stereotactic radiosurgery, long-term outcomes, matched cohort analyses

## Abstract

**Simple Summary:**

Intracranial schwannomas are benign intracranial neoplasms. Vestibular schwannomas (VS) account for 90% of intracranial schwannomas; whereas the other cranial nerve schwannomas, such as trigeminal (TS), facial (FS), and jugular foramen schwannoma (JFS), account for 10% of all cases. Stereotactic radiosurgery (SRS) is a minimally invasive treatment for small to medium VS and known to provide excellent long-term tumor control; however, there remains a paucity of evidence regarding SRS for TS, FS, and JFS due to the rarity of these tumors. We investigated the radiosurgical outcomes of these non-vestibular schwannomas and compared them to those of VS through matched cohort analysis.

**Abstract:**

Stereotactic radiosurgery (SRS) is known to provide excellent tumor control with functional preservation for vestibular schwannomas (VS), but its efficacy in the other major intracranial schwannomas including trigeminal (TS), facial (FS), and jugular foramen schwannomas (JFS) has not been established yet due to their rarity. We retrospectively analyzed data of 514 consecutive patients who had intracranial schwannomas (460 VS, 22 TS, 7 FS, and 25 JFS) and underwent SRS. The 5- and 10-year tumor control rates were 97% and 94% for VS, 100% and 100% for TS, 80% and 80% for FS, and 100% and 80% for JFS. Radiation-induced complications included one hydrocephalus for TS (4.5%), no cases for FS (0%), and one hydrocephalus and one lower palsy for JFS (8.0%). Through matched cohort analysis between patients with VS and each of the non-VS, we found no statistical difference in tumor control and radiation-induced adverse events. SRS seems to provide long-term tumor control with functional preservation for TS, FS and JFS and the efficacies are similar to VS.

## 1. Introduction

Intracranial schwannomas are the third most common benign neoplasms that are thought to arise from Schwann cells and account for 6–12.3% of all intracranial tumors [1,2,3]. While vestibular schwannoma (VS) accounts for 90% of intracranial schwannomas, trigeminal schwannoma (TS), facial schwannoma (FS), and jugular foramen schwannoma (JFS) are rare and account for 10% of them [4,5]. Patients generally present with symptoms related to the affected cranial nerves, such as hearing loss in VS, facial dysesthesia/paresthesia in TS, facial paralysis in FS, and dysphagia/dysphonia in JFS, which lead to a significant decline in the quality of life [6,7,8,9,10]. Surgical resection is a standard treatment for non-VS, however, the procedures are not always easy due to the deep skull base locations of these tumors and the necessity of functional preservation.

Stereotactic radiosurgery (SRS) is a minimally invasive treatment option for small to medium VS and provides an approximately 90% rate of favorable tumor control over 10 years [11,12,13,14]. Given the robust evidence demonstrating favorable long-term tumor control for VS and the similar cellular origin and benignity, SRS is expected to provide a similar level of efficacy for TS, FS, and JFS [15]. Nevertheless, although several studies have described the outcomes of SRS for non-VS, the numbers of patients are limited, and thus those studies may be susceptible to biases, leading to suboptimal external validity [2,6,9,16,17,18,19,20]. To compensate for the lack of patient numbers in single-center studies, a few multicenter studies have been conducted [9,21,22]. However, in multicenter studies for such rare tumors, inter-hospital diversities in treatment policies and radiosurgical doses might hinder the outcomes and interpretation. Additionally, these studies may not be enough to address long-term outcomes. Therefore, there remains a paucity of data on the long-term efficacy of SRS for TS, FS, and JFS.

We have performed SRS for these non-VS and obtained the long follow-up data. Nevertheless, the patient numbers were not as large as the numbers in multicenter studies; therefore, a simple descriptive analysis cannot eliminate the selection bias. One alternative approach to address this issue would be to compare the outcomes to those of VS, which have been well-established. Therefore, the goal of this study was to confirm the long-term efficacy and clarify the role of SRS for TS, FS, and JFS through statistical comparisons to VS.

## 2. Results

### 2.1. Baseline Characteristics of the Entire Group

Patient characteristics are shown in Table 1. Briefly, 514 patients were included in this study (460 VS, 22 TS, 7 FS, and 25 JFS). The median observation periods (±standard deviation) were 103 ± 92 months for VS, 82 ± 80 for TS, 86 ± 50 for FS and 58 ± 54 for JFS, respectively. The observation period was significantly shorter in the JFS group than the VS group. Target volume was significantly larger in the TS and JFS group than the VS group (*p* < 0.001), and radiosurgical doses were significantly higher in the TS (prescription dose: *p* = 0.001; central dose: *p* = 0.039) and JFS group (prescription dose: *p* = 0.002; central dose: *p* = 0.002). The JFS group had a more surgical history than the VS group (*p* < 0.001). 

### 2.2. Endpoints for the Entire Cohort

**Figure 1:**
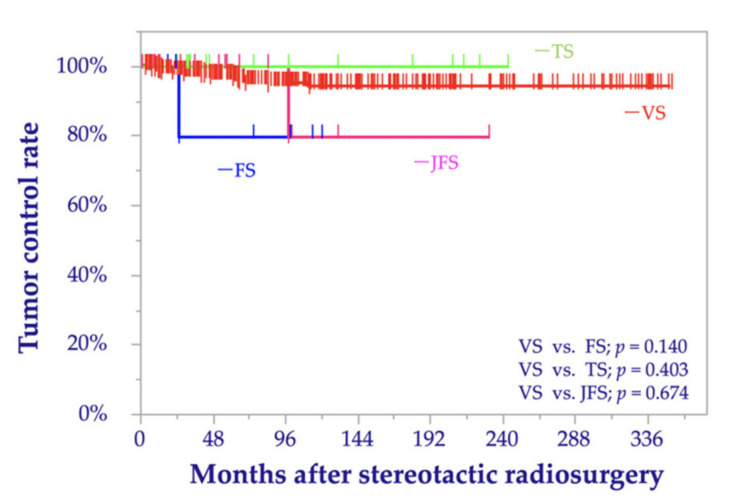
Kaplan–Meier curves of the tumor control rates before matching comparing trigeminal schwannoma (TS), facial schwannoma (FS), jugular foramen schwannoma (JFS), and vestibular schwannoma (VS).

In the entire cohort, one tumor progression following SRS was confirmed in the FS group (14.3%), one in the JFS group (4.0%), and 16 in the VS group (3.5%). No tumor progression was observed in the TS group. The 10-year tumor control rates (TCR) were 100% for TS, 80% for FS, 80% for JFS, and 94% for VS. The log-rank test showed no significant differences among the tumor groups (TS vs. VS: *p* = 0.403; FS vs. VS: *p* = 0.140; and JFS vs. VS: *p* = 0.674) (Figure 1). 

No factors were significantly associated with tumor progression in the bivariate and multivariable Cox proportional hazard analyses (Table 2).

Throughout the observation period, any radiation-induced adverse events (RAEs) were observed in one patient (4.5%) in the TS group, no patients in the FS group, two patients (8.0%) in the JFS group, and 39 patients (8.5%) in the VS group. Among them, one patient (4.5%) with TS, one patient (4.0%) with JFS, and 23 (5.0%) with VS had Common Terminology Criteria for Adverse Events (CTCAE) grade 3 or 4 RAEs (Table 3). In particular, the FS group included five patients without facial palsy, one with House and Blackman grade III facial palsy, and the other with grade IV facial palsy. All the patients without facial palsy maintained facial nerve function, whereas there was no improvement in cranial nerve function among two patients with facial palsy during the study period.

A significant difference was observed in the lower palsy as a specific adverse event of JFS (*p* = 0.001), but no other significant differences in the rates of RAEs were observed between the cohorts. According to the bivariate analysis, the prescribed dose of >12 Gy (odds ratio [OR], 7.79; 95% confidence interval [CI], 3.1–19.5; *p* < 0.001) and central dose of >25 Gy (OR, 4.04; 95% CI, 1.6–10.1; *p* = 0.003) were significantly associated with cranial nerve (CN) injuries, and only a prescription dose > 12 Gy remained significant in the multivariable analysis (OR, 8.67; 95% CI, 3.4–22.3; *p* < 0.001) (Table 4).

### 2.3. Matched Cohort Analysis and Patient Background of the Matched Cohort

A total of 22 TS, 7 FS, 25 JFS, and 460 VS patients were eligible for matching. Matching was performed by a statistician who was blinded to the treatment results (T.S.). One-to-one coarsened exact matching based on age, sex, tumor volume, and surgical history yielded three VS cohorts comprising of the same numbers of patients as the TS, FS, and JFS cohorts. Further analyses were performed in three pairs of cohorts comprising of 19 TS and 19 VS patients (mean ± standard deviation [SD] observation periods of 50.1 ± 15.4 months and 50.5 ± 14.7 months, respectively), 7 FS and 7 VS patients (61.6 ± 12.7 months and 60.6 ± 4.9 months, respectively), and 18 JFS and 18 VS patients (47.9 ± 18.6 months and 48.3 ± 16.4 months, respectively). This exact matching process resulted in the exclusion of three patients with TS and seven patients with JFS from matched cohorts. The comparisons of the patients’ backgrounds are summarized in Tables S1–S3.

### 2.4. Endpoints in the Matched Cohorts

**Figure 2:**
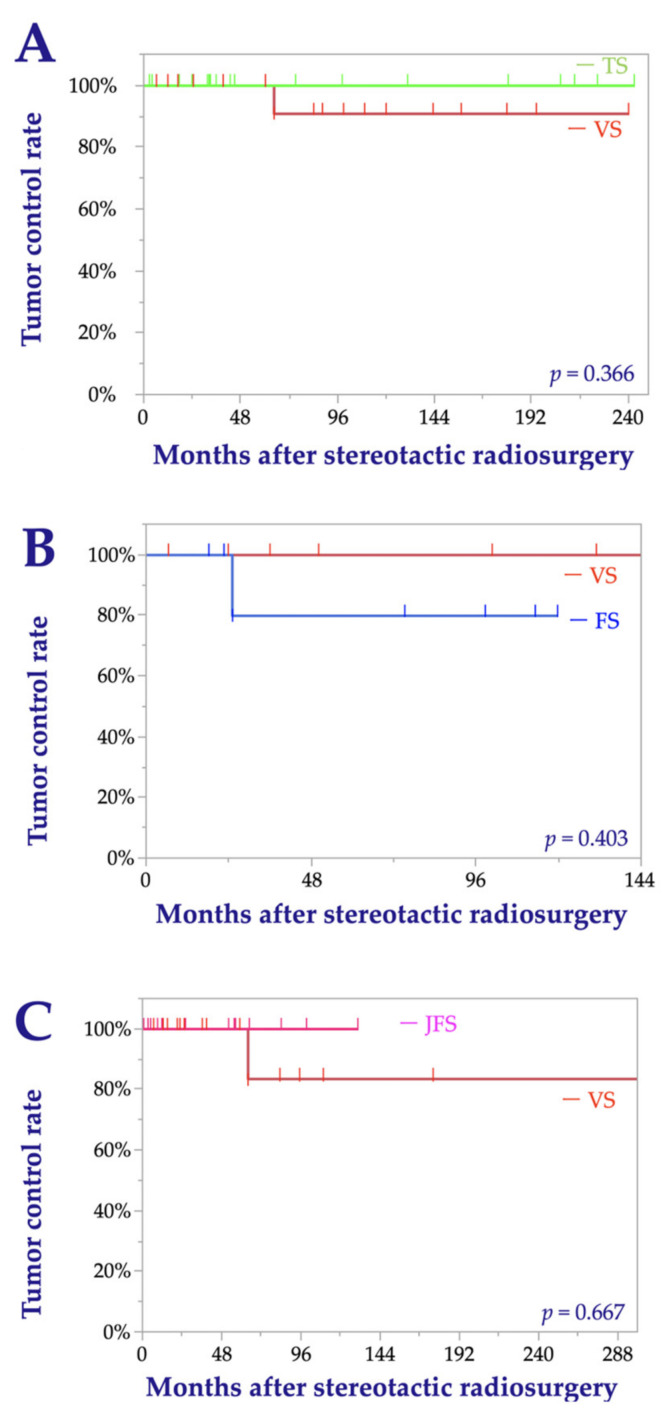
Kaplan–Meier curves for the tumor control rates after matching that compare trigeminal schwannoma (TS) and vestibular schwannoma (VS) (**A**), facial schwannoma (FS) and VS (**B**), and jugular foramen schwannoma (JFS) and VS (**C**).

Among the TS cohort, tumor progression was not observed after SRS, resulting in a TCR of 100% at 10 to 20 years; among the pertinent VS cohort, one (5.3%) tumor showed progression, yielding a TCR of 91% at 10 to 20 years (log-rank test; *p* = 0.366) (Figure 2A). Among the FS cohort, one (14.3%) tumor showed progression after SRS, resulting in a TCR of 80% at 5 to 10 years; among the pertinent VS cohort, no tumors showed progression, resulting in a TCR of 100% at 5 to 10 years (log-rank test; *p* = 0.403) (Figure 2B). Among the JFS cohort, no tumor showed progression after SRS, resulting in a TCR of 100% at 5 to 10 years. Among the pertinent VS cohort, one (5.6%) tumor showed progression, resulting in a TCR of 100% at 5 years and 83% at 10 years (log-rank test; *p* = 0.667) (Figure 2C).

Regarding RAEs, in the matched pair cohorts pertinent to TS, there was one case (5.3%) with one RAE (hydrocephalus of CTCAE grade ≥ 3) in the TS cohort and one case (5.3%) with two RAEs (facial palsy and hydrocephalus of CTCAE grade ≥ 3) in the VS cohort. In the matched pair cohorts pertinent to FS, no RAEs were observed. In the matched pair cohorts pertinent to JFS, there was one case (5.6%) with one RAE (hydrocephalus) in the JFS cohort and two cases (11.1%) with RAEs (one had facial palsy and hydrocephalus of CTCAE grade ≥ 3, and the other had hydrocephalus of CTCAE grade ≥ 3) in the VS cohort. Between any pairs of cohorts, no significant differences were confirmed in the incidences of any complications (Tables S4–S6).

## 3. Discussion

In the present study, we examined the long-term outcomes of SRS for TS, FS, and JFS through the multivariable analyses and matched cohort analyses in comparisons to VS, for which SRS is known to be highly effective. We demonstrated that SRS for TS, FS, and JFS appeared to provide favorable tumor control that is similar to VS with acceptably low-risk profiles.

### 3.1. The Role of SRS for TS

Surgical resection is the standard treatment for large and symptomatic intracranial schwannomas because it provides immediate and significant decompression [23,24,25]. TS originates in the Gasserian ganglion within the middle fossa and often extends into the cavernous sinus, posterior fossa, orbit, pterygopalatine fossa, and infratemporal fossa, and involves the neurovascular structures of the skull base. Various surgical approaches including transmaxillary, orbitozygomatic, subtemporal, retrosigmoid suprameatal, presigmoid, and recently endoscopic transnasal approaches have been reported. Nevertheless, complete resection may not be always achievable, and is associated with a 15% to 59.6% risk of CN dysfunction and a 0 to 17% recurrence rate [20,23,26,27,28].

As for radiosurgical outcomes of TS, there are 18 studies involving 8 to 74 patients (a total of 531 cases) to date [19,20,29,30], demonstrating 77% to 100% crude rates of tumor control and 6% to 37% rates of RAEs. Additionally, facial paresthesia, which occurs in the majority of patients with TS, has been reported to improve with SRS in 47% of patients and to worsen in 21% of patients [2]. Neurofibromatosis type 2 (NF2)-related tumor is reported to be associated with failed tumor control. Our 22 patients with TS were treated with a mean prescription dose of 13.8 Gy, resulting in an excellent TCR of 100% (crude rate) and a low RAE rate of 4.5%. Based on the matched-cohort analysis that demonstrated a similar level of efficacy to VS, SRS for TS is deemed as reliable and reasonable as SRS for VS.

### 3.2. The Role of SRS for FS

FS is much rarer than TS and JFS. The most common location is the geniculate ganglion (44%), followed by the tympanic segment (43%), vertical segment (37%), canalicular segment (24%), and CPA (18%). Treatment options for FS should be discussed in terms of resectability, size and location, and functional outcomes, and patients should be informed of the risk of various complications. Although various approaches such as translabyrinthine, transmastoid, and retrosigmoid are effective, if the tumor is not sufficiently detached from the facial nerve, the risk of recurrence could be relatively high. Additionally, the post-operative facial nerve deterioration and atrophy could be troublesome. If part of the facial nerve needs to be resected, a nerve graft or in situ nerve anastomosis is required; however, it is challenging to maintain facial nerve function even with such techniques [7,21,24,31,32]. In a surgical series of 53 cases of FS, it was reported that facial nerve sacrifice was necessary for 67.9% of patients; in those patients with anatomically preserved nerves, 23.5% had postoperative House and Brackmann grade 4 or higher facial function [32]. Additionally, McMonagle et al. recommended waiting for the House and Brackmann grade to deteriorate to 3 or 4 before FS resection because of the high risk of postoperative facial nerve injury [33].

Regarding SRS, 150 cases have been reported in 12 studies involving 1 to 63 cases [21,31,32,34], with TCR of 83% to 100% (crude rate). Facial weakness may improve following SRS in 47–60% or decline in 12.8–21% [2]. For our seven patients with a mean prescription dose of 12.9 Gy, the TCR was 86% (crude rate) and no patient experienced a decline in facial nerve function. The treatment results were also comparable with those of VS after matching.

### 3.3. The Role of SRS for JFS

The treatment strategy of JFS and associated risks mainly depends on anatomic and tumor-growth characteristics. Surgical management of JFS remains challenging due to many of these anatomical features, including the deep location, surrounding neurovascular structures such as the lower cranial nerves, carotid artery, jugular vein unless occluded, and brainstem, and frequent intra- and extracranial involvement [35,36]. A variety of modified surgical approaches have been recommended for the management of JFS, including the retrosigmoid approach for intracranial type, infratemporal fossa approach for extracranial type, a combination of these, or translabyrinthine, transcochlear, transjugular, and transsigmoid for intraosseous and combined types [8,35,36,37,38,39,40,41,42]. With JFS, gross total or near-total resection may be achievable in approximately two-thirds of cases; however, this is difficult to achieve for the combined intra- and extracranial type, and postoperative CN morbidity is high (lower CN morbidity, 6–100%; facial nerve morbidity, 11–80%; vestibular and acoustic nerve morbidity, 4–45%). Depending on the severity of the morbidity, additional procedures, such as a nasogastric tube, percutaneous endoscopic gastronomy, vocal cord injection, and tracheostomy may be necessary. In contrast, postoperative improvement can be expected in more than 50% of patients [41,42,43]. Park et al. reported that subtotal resection with SRS aimed at functional preservation improves the TCR, symptoms, and postoperative neurological deficits compared with radical resection. Subtotal resection with the SRS group achieved significantly lower postoperative CN morbidity, especially with respect to dysphagia and related functional state, and excellent tumor control (9 cases, 100%) with a mean observation period of 34 months [43]. Notwithstanding, there might remain concerns about future recurrence with this method.

Reports on SRS for JFS are still limited. Two studies involving 92 patients and 117 patients have reported TCR of 87% to 89% at 5 years and RAE rates of 7% to 15% [9,22]. Factors associated with failed tumor control include dumbbell-type tumors, brainstem edema, and tumor volume >6 cm^3^. The treatment outcomes of our 25 patients treated with a mean prescription dose of 13.5 Gy resulted in excellent TCR of 100% at 5 years and 80% at 10 years, with an RAE rate of 8%. The treatment results after matching were sufficiently equivalent to those of VS.

In summary, given the excellent tumor control and acceptable low risk of neurological deterioration following SRS, SRS would be effective and safe for TS, FS, and JFS, and thus a reasonable alternative to surgical resection for small- to medium-sized tumors. There were several limitations to the present study. First, this study was a single-institution, retrospective analysis. A prospective study with more cases and a longer follow-up period is desirable to reduce selection bias. Second, the number of patients with VS and the number of patients with TS, FS, and JFS were significantly different, which might have affected the statistical analyses of these cohorts. To correct this influence, we performed one-to-one coarsened exact matching to confirm the consistency of results; however, the existence of significant differences in some other variables could not be denied after matching. Third, the sample size was not so large; we will need to conduct further analysis with a larger sample size to verify that the prognosis is truly similar among the type of tumor.

## 4. Materials and Methods

### 4.1. Study Population

We retrospectively collected data on 563 consecutive patients with intracranial schwannomas treated with SRS at our institution from June 1990 to May 2020. A total of 46 cases without sufficient follow-up data (less than 1 month) were excluded. Additionally, there were 3 other schwannomas (1 oculomotor and 2 abducens), which were excluded from the cohort as the numbers were too small for statistical analysis.

### 4.2. Radiosurgical Techniques, Treatment Indications and Management After SRS

The Leksell Gamma Knife (Elekta AB, Stockholm, Sweden) was used to deliver the SRS treatments. Stereotactic imaging (CT before July 1996, MRI between August 1996 and January 2018, MRI and cone-beam CT thereafter) was performed to obtain precise data regarding the shape, volume, and three-dimensional coordinates of tumors after head fixation using a Leksell frame (Elekta Instruments, Stockholm, Sweden). Then, a radiosurgical treatment plan was created and approved by dedicated neurosurgeons and radiation oncologists. All treatment plans were performed on a commercially available software (KULA treatment planning system until 1998 and Leksell GammaPlan^®^ [Elekta Instruments] thereafter).

Treatment strategies for each tumor were discussed and approved at conferences held in the Department of Neurosurgery. In principle, surgical resection was recommended for large tumors (maximum diameter >25–30 mm) regardless of patients’ age and for medium to large tumors in young patients (<40 years-old). Conservative therapy with annual radiological follow-up was considered for small asymptomatic tumors.

Following SRS, we performed follow-up MRIs as well as clinical evaluations every 6 months, data of which were prospectively collected to our treatment database. The radiologic findings were independently assessed by neurosurgeons and radiologists. After confirming that the treated tumor appeared to be stable over the first 3 years following SRS, the follow-up interval was extended to 1 year. Transient expansion, which is commonly observed in VS following SRS and thought to represent radiation-induced tumor swelling rather than essential tumor growth, was judged carefully to avoid confusion with recurrence after due consideration of the findings of serial MRIs [24].

### 4.3. Statistical Analysis

The baseline characteristics of all patients were summarized. To examine the therapeutic effects, the TCR were calculated using the Kaplan–Meier method and compared between VS, TS, FS, and JFS cohorts using the log-rank test. The therapeutic outcomes were labeled as “under control” or “treatment failure,” depending on whether additional therapeutic interventions were required. Normally, tumor recurrence is defined by an increase in size [44,45,46,47]. In the present study, we defined treatment failure as described above to avoid counting transient tumor expansion. Clinical factors associated with tumor control were examined with bivariate and multivariable Cox proportional analyses. The RAEs were graded according to CTCAE version 5.0 and compared among the tumor groups using Pearson’s chi-square test. Factors associated with RAE were examined using logistic regression analysis. The TCR and RAE rates were evaluated for each tumor. Continuous variables (age, target volume, and radiosurgical doses) were entered into models after being dichotomized using mean values. Multivariable analyses including the presence of NF2, age at the time of SRS, tumor volume, prescription dose, sex, surgical history, and schwannoma type were performed.

As significant differences in the background characteristics of patients with VS and the other intracranial schwannomas were expected, we performed matching to correct for the variability of baseline characteristics. Due to the small numbers in TS, FS, and JFS, we performed one-to-one coarsened exact matching based on age, sex, tumor volume, and surgical history to match the baseline characteristics related to the outcome of SRS as closely as possible. As a result, three VS cohorts with the same numbers of patients as the TS, FS, and JFS cohorts was created. One-to-one coarsened exact matching without replacement was performed for age at the time of SRS, sex (categorical), volume, and history of surgery (categorical). We conducted the same bivariate analyses of TCR and RAE for the matched groups. All statistical analyses were performed using JMP^®^ Pro 15.0.0 software (SAS Institute Inc., Cary, NC, USA) and Stata 15.1 (StataCorp, College Station, TX, USA).

## 5. Conclusions

SRS results in excellent long-term tumor control and minimal invasiveness for TS, FS, and JFS. Considering the difficulty in achieving tumor control by surgery alone and the high post-operative morbidity rate, SRS may be an effective and safe modality for TS, FS, and JFS and a reasonable alternative to surgical resection for small- to medium-sized intracranial schwannomas.

## Figures and Tables

**Table 1:** Baseline characteristics and dosimetry data of the entire cohort before matching.

Variables	VS	TS	FS	JFS	*p*-Value (TS)	*p*-Value (FS)	*p*-Value (JFS)
					Reference: VS		
Patients (tumors)	460	22	7	25			
Age at SRS, years	55.0 ± 14.1	48.2 ± 17.5	61.6 ± 12.6	49.1 ± 18.9	0.070	0.248	0.069
Observation period, months	103.2 ± 91.8	82.0 ± 80.3	85.5 ± 49.9	58.4 ± 53.5	0.221	0.992	0.022 *
Target volume, cm^3^	2.1 ± 2.3	5.1 ± 4.2	1.7 ± 1.9	5.7 ± 4.5	<0.001 *	0.374	<0.001 *
Prescription dose, Gy	13.1 ± 2.0	13.8 ± 1.6	12.9 ± 2.3	13.5 ± 1.0	0.001 *	0.443	0.002 *
Central dose, Gy	26.7 ± 4.0	28.7 ± 5.0	25.3 ± 6.1	28.5 ± 3.1	0.039 *	0.319	0.002 *
Male	222 (48.3)	10 (45.5)	2 (28.6)	13 (54.2)	0.797	0.301	0.451
Surgical history	107 (23.3)	6 (27.3)	3 (42.9)	14 (58.3)	0.664	0.225	<0.001 *

* < 0.05 is considered statistically significant. Data are reported as mean ± standard deviation or n (%) unless otherwise stated. VS, vestibular schwannoma; TS, trigeminal schwannoma; FS, facial schwannoma; JFS, jugular foramen schwannoma; SRS, stereotactic radiosurgery.

**Table 2:** Results of bivariate and multivariable analyses of tumor progression before matching.

Variables	Bivariate		Multivariable	
	*p*-Value	HR (95% CI)	*p*-Value	HR (95% CI)
Presence of NF2 (ref. absence)	0.999	0.01 (0.00–*)	/	/
Age at SRS > 55 years (ref. ≤ 55 years)	0.441	1.43 (0.58–3.56)	0.666	1.24 (0.47–3.25)
Volume > 1.5 cm^3^ (ref. ≤ 1.5 cm^3^)	0.594	0.78 (0.32–1.93)	0.628	0.80 (0.32–1.99)
Prescription dose > 12 Gy (ref. ≤ 12 Gy)	0.918	1.05 (0.42–2.62)	0.900	1.07 (0.41–2.77)
Central dose > 25 Gy (ref. ≤ 25 Gy)	0.609	0.78 (0.31–2.00)	/	/
Male (ref. female)	0.656	1.23 (0.49–3.06)	/	/
History of surgery (ref. no history of surgery)	0.060	0.41 (0.17–1.04)	0.144	0.49 (0.19–1.28)
Tumor type (ref. VS)				
TS	0.999	0.01 (0.00–*)	0.999	0.01 (0.00–*)
FS	0.192	0.26 (0.03–1.96)	0.266	0.31 (0.04–2.45)
JFS	0.685	0.66 (0.09–4.97)	0.926	0.91 (0.12–7.14)

* Values could not be calculated because tumor progression was not observed in this cohort. HR, hazard ratio; CI, confidence interval; NF2, neurofibromatosis type 2; ref., reference; SRS, stereotactic radiosurgery; VS, vestibular schwannoma; TS, trigeminal schwannoma; FS, facial schwannoma; JFS, jugular foramen schwannoma.

**Table 3:** Summary of radiation-induced adverse events after SRS before matching.

Variables	VS	TS	FS	JFS	*p*-Value
All complications, n (%)	39 (8.5)	1 (4.5)	0 (0.0)	2 (8.0)	0.785
Trigeminal neuralgia, n (%)	12 (2.6)	0 (0.0)	0 (0.0)	0 (0.0)	0.702
Facial palsy, n (%)	21 (4.6)	0 (0.0)	0 (0.0)	0 (0.0)	0.471
Lower palsy, n (%)	0 (0.0)	0 (0.0)	0 (0.0)	1 (4.0)	0.001 *
Hydrocephalus, n (%)	17 (3.7)	1 (4.5)	0 (0.0)	1 (4.0)	0.769
CTCAE grade 3–4, n (%)	23 (5.0)	1 (4.5)	0 (0.0)	1 (4.0)	0.936

* *p* < 0.05 is considered statistically significant. VS, vestibular schwannoma; TS, trigeminal schwannoma; FS, facial schwannoma; JFS, jugular foramen schwannoma; CTCAE, Common Terminology Criteria for Adverse Events version 5.0.

**Table 4:** Results of bivariate and multivariable analyses of cranial nerve injuries considered radiation-related adverse events before matching.

Variables	Bivariate		Multivariable	
	*p*-Value	OR (95% CI)	*p*-Value	OR (95% CI)
Presence of NF2 (ref. absence)	0.985	1.01 (0.2–4.5)	0.955	0.96 (0.2–4.6)
Age at SRS > 55 years (ref. ≤ 55 years)	0.870	1.07 (0.5–2.3)	0.189	1.72 (0.8–3.9)
Volume > 1.5 cm^3^ (ref. ≤ 1.5 cm^3^)	0.087	1.99 (0.9–4.4)	0.132	1.88 (0.8–4.3)
Prescription dose > 12 Gy (ref. ≤ 12 Gy)	<0.001 *	7.79 (3.1–19.5)	<0.001 *	8.67 (3.4–22.3)
Central dose > 25 Gy (ref. ≤ 25 Gy)	0.003 *	4.04 (1.6–10.1)	/	/
Male (ref. female)	0.132	0.55 (0.8–4.0)	/	/
History of surgery (ref. no history of surgery)	0.543	0.75 (0.3–1.9)	0.426	0.67 (0.3–1.8)
Tumor type (ref. VS)				
TS	0.990	0.01 (0.0–**)	/	/
FS	0.994	0.01 (0.0–**)	/	/
JFS	0.671	0.64 (0.1–4.9)	/	/

* *p* < 0.05 is considered statistically significant. ** Values could not be calculated because cranial nerve injuries were not observed in these cohorts. OR, odds ratio; CI, confidence interval; NF2, neurofibromatosis type 2; ref., reference; SRS, stereotactic radiosurgery.

## Data Availability

The data presented in this study are available on request from the corresponding author.

## Supplementary Materials

The following are available online at https://www.mdpi.com/2072-6694/13/5/1140/s1, Tables S1–S6 were included as digital contents. Table S1. Baseline characteristics and dosimetry data of the TS-matched cohort. Data are reported as mean ± standard deviation or n (%) unless otherwise stated. VS, vestibular schwannoma; TS, trigeminal schwannoma; SRS, stereotactic radiosurgery. Table S2. Baseline characteristics and dosimetry data of the FS-matched cohort. Data are reported as mean ± standard deviation or n (%) unless otherwise stated. VS, vestibular schwannoma; FS, facial schwannoma; SRS, stereotactic radiosurgery. Table S3. Baseline characteristics and dosimetry data of the JFS-matched cohort. * *p* < 0.05 is considered statistically significant. Data are reported as mean ± standard deviation or n (%) unless otherwise stated. VS, vestibular schwannoma; JFS, jugular foramen schwannoma; SRS, stereotactic radiosurgery. Table S4. Summary of radiation-induced adverse events after SRS in TS matched cohort. All events occurred in the same case. VS, vestibular schwannoma; TS, trigeminal schwannoma; RAEs; radiation-induced adverse events; CTCAE, Common Terminology Criteria for Adverse Events version 5.0; N/A, not applicable. Table S5. Summary of radiation-induced adverse events after SRS in FS matched cohort. VS, vestibular schwannoma; FS, facial schwannoma; RAEs; radiation-induced adverse events; CTCAE, Common Terminology Criteria for Adverse Events version 5.0; N/A, not applicable. Table S6. Summary of radiation-induced adverse events after SRS in JFS matched cohort. VS, vestibular schwannoma; JFS, jugular foramen schwannoma; RAEs; radiation-induced adverse events; CTCAE, Common Terminology Criteria for Adverse Events version 5.0; N/A, not applicable.
