# Supplementary material for: Long-Term Outcomes of Stereotactic Radiosurgery for Trigeminal, Facial, and Jugular Foramen Schwannoma in Comparison with Vestibular Schwannoma
Source: Cancers (Basel). 2021 Mar 7;13(5):1140. doi: 10.3390/cancers13051140 (PMC7961475; doi:10.3390/cancers13051140)
Supplement: Supplementary file 1 [file cancers-13-01140-s001.pdf]

Table S1. Baseline characteristics and dosimetry data of the TS-matched cohort.

Variables	VS	TS	*p*-value
Patients (tumors), n	19	19	
Age at SRS, years	50.5 ± 14.7	50.1 ± 15.4	0.930
Observation period, months	102.8 ± 87.3	91.5 ± 82.4	0.737
Target volume, cm^3	4.0 ± 2.5	3.9 ± 2.4	0.930
Prescription dose, Gy	13.7 ± 2.4	14.1 ± 1.5	0.141
Central dose, Gy	28.3 ± 4.6	29.4 ± 5.0	0.403
Male	8 (42.1)	8 (42.1)	1.000
Surgical history	3 (15.7)	3 (15.7)	1.000

Data are reported as mean ± standard deviation or n (%) unless otherwise stated. VS, vestibular schwannoma; TS, trigeminal schwannoma; SRS, stereotactic radiosurgery.

Table S2. Baseline characteristics and dosimetry data of the FS-matched cohort.

Variables	VS	FS	*p*-value
Patients (tumors), n	7	7	
Age at SRS, years	60.6 ± 4.9	61.6 ± 12.7	0.949
Observation period, months	72.5 ± 58.2	85.5 ± 49.9	0.848
Target volume, cm^3	1.7 ± 1.9	1.7 ± 1.9	0.898
Prescription dose, Gy	12.6 ± 1.0	12.9 ± 2.3	0.656
Central dose, Gy	26.0 ± 2.6	25.3 ± 6.1	0.494
Male	2 (28.6)	2 (28.6)	1.000
Surgical history	3 (42.9)	3 (42.9)	1.000

Data are reported as mean ± standard deviation or n (%) unless otherwise stated. VS, vestibular schwannoma; FS, facial schwannoma; SRS, stereotactic radiosurgery.

Table S3. Baseline characteristics and dosimetry data of the JFS-matched cohort.

Variables	VS	JFS	*p*-value
Patients (tumors), n	18	18	
Age at SRS, years	48.3 ± 16.4	47.9 ± 18.6	0.825
Observation period, months	79.5 ± 96.4	43.9 ± 37.0	0.311
Target volume, cm^3	4.3 ± 5.3	4.1 ± 3.7	0.849
Prescription dose, Gy	13.2 ± 2.3	13.6 ± 1.0	0.038*
Central dose, Gy	27.1 ± 4.9	28.9 ± 3.3	0.098
Male	8 (44.4)	8 (44.4)	1.000
Surgical history	11 (61.1)	11 (61.1)	1.000

* $p < 0.05$ is considered statistically significant. Data are reported as mean ± standard deviation or n (%) unless otherwise stated. VS, vestibular schwannoma; JFS, jugular foramen schwannoma; SRS, stereotactic radiosurgery.

Table S4. Summary of radiation-induced adverse events after SRS in TS matched cohort.

Variables	VS	TS	*p*-value
Number of cases with RAEs	1 (5.3)	1 (5.3)	1.000
Trigeminal neuralgia	0 (0.0)	0 (0.0)	N/A
Facial palsy	1 (5.3)	0 (0.0)	0.311
Lower palsy	0 (0.0)	0 (0.0)	N/A
Hydrocephalus	1 (5.3)	1 (5.3)	1.000
CTCAE grade 3–4	1 (5.3)	1 (5.3)	1.000

All events occurred in the same case. VS, vestibular schwannoma; TS, trigeminal schwannoma; RAEs; radiation-induced adverse events; CTCAE, Common Terminology Criteria for Adverse Events version 5.0; N/A, not applicable.

Table S5. Summary of radiation-induced adverse events after SRS in FS matched cohort.

Variables	VS	FS	*p*-value
Number of cases with RAEs	0 (0.0)	0 (0.0)	N/A
Trigeminal neuralgia	0 (0.0)	0 (0.0)	N/A
Facial palsy	0 (0.0)	0 (0.0)	N/A
Lower palsy	0 (0.0)	0 (0.0)	N/A
Hydrocephalus	0 (0.0)	0 (0.0)	N/A
CTCAE grade 3–4	0 (0.0)	0 (0.0)	N/A

VS, vestibular schwannoma; FS, facial schwannoma; RAEs; radiation-induced adverse events; CTCAE, Common Terminology Criteria for Adverse Events version 5.0; N/A, not applicable.

Table S6. Summary of radiation-induced adverse events after SRS in JFS matched cohort.

Variables	VS	JFS	*p* value
Number of cases with RAEs	2 (11.1)	1 (5.6)	0.547
Trigeminal neuralgia	0 (0.0)	0 (0.0)	N/A
Facial palsy	1 (5.6)	0 (0.0)	0.311
Lower palsy	0 (0.0)	0 (0.0)	N/A
Hydrocephalus	2 (11.1)	1 (5.6)	0.547
CTCAE grade 3–4	2 (11.1)	0 (0.0)	0.146

VS, vestibular schwannoma; JFS, jugular foramen schwannoma; RAEs; radiation-induced adverse events; CTCAE, Common Terminology Criteria for Adverse Events version 5.0; N/A, not applicable.
